# As the SARS-CoV-2 virus evolves, should Omicron subvariant BA.2 be subjected to quarantine, or should we learn to live with it?

**DOI:** 10.3389/fpubh.2022.1039123

**Published:** 2022-11-24

**Authors:** Ren Xu, Wanning Wang, Wenlong Zhang

**Affiliations:** ^1^Pulmonary and Critical Care Medicine Department, First Hospital of Jilin University, Changchun, China; ^2^Nephrology Department, First Hospital of Jilin University, Changchun, China; ^3^Department of Hematology and Oncology, China-Japan Union Hospital of Jilin University, Changchun, China

**Keywords:** COVID-19, SARS-CoV-2, Omicron BA.2, quarantine, evolve

## Abstract

It has been nearly 35 months since the COVID-19 outbreak. The pathogen SARS-CoV-2 has evolved into several variants. Among them, Omicron is the fifth variant of concern which have rapidly spread globally during the past 8 months. Omicron variant shows different characteristics from previous variants, which is highly infectious, highly transmissible, minimally pathogenic, vaccine and antibody tolerant; however, it is less likely to cause severe illness, resulting in fewer deaths. Omicron has evolved into five main lineages, including BA.1, BA.2, BA.3, BA.4, and BA.5. Before BA.5, Omicron BA.2 sublineage was the dominant strain all over the world for several months. The experience of prevention and treatment against BA.2 is worth studying and learning for overcoming other Omicron subvariants. Although the Omicron subvariant BA.2 is significantly less severe than that caused by ancestral strains, it is still far more dangerous than influenza, and its long-term sequelae are unknown. Effective treatments are currently limited; therefore, effective defense may be the key to controlling the epidemic today, rather than just “living with” the virus.

## Introduction

**Figure 1:**
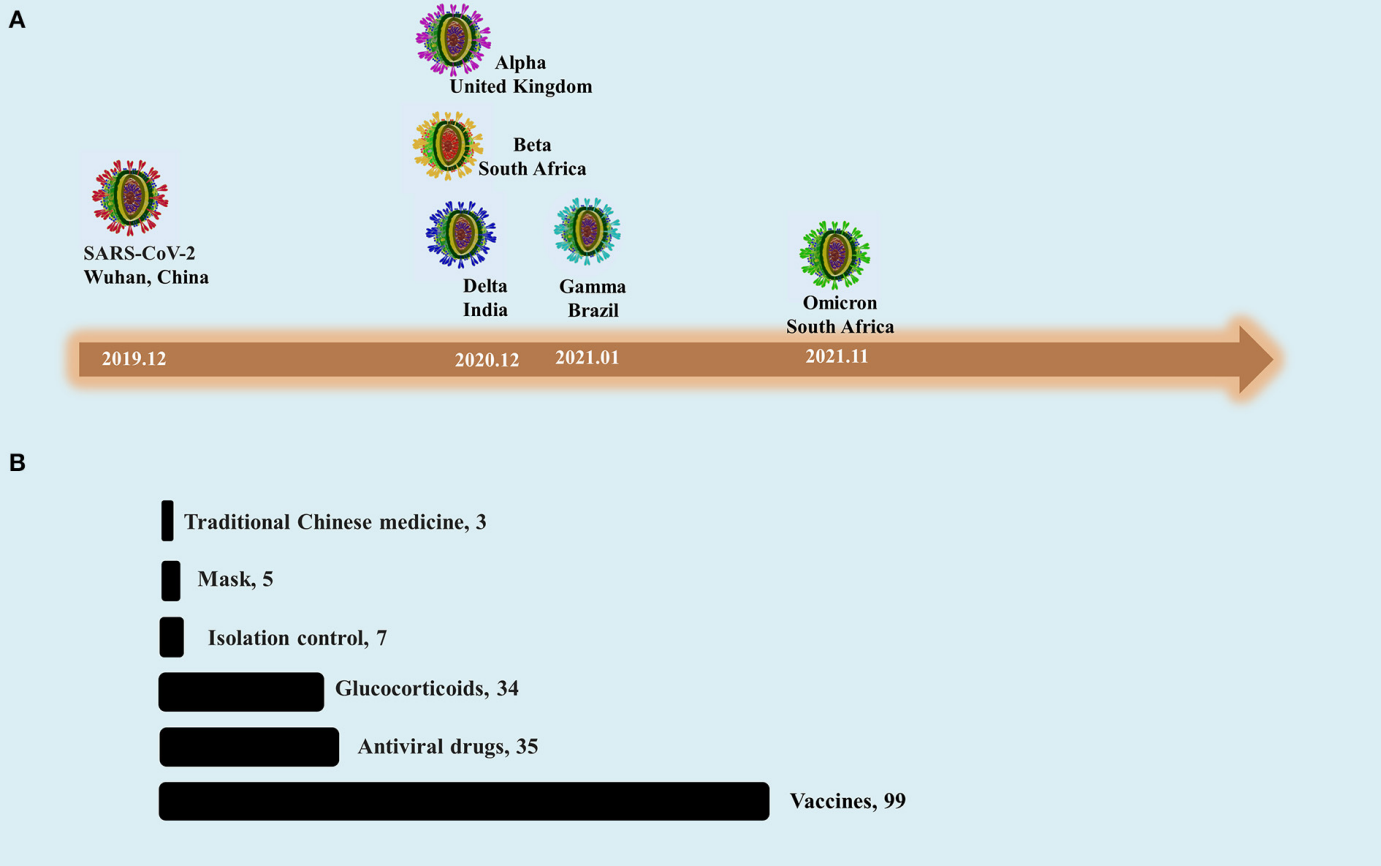
The timeline of SARS-CoV-2 evolution and hot spots in prevention and therapeutic measures research. **(A)** The VOCs of SARS-CoV-2 and their first reported or identified timepoints and regions. **(B)** Numbers of clinical studies retrieved from PUBMED official website. Strategies to treat with COVID-19 and its variants include vaccines, antiviral drugs, glucocorticoids, isolation control, masks and traditional Chinese medicine, etc. The internal factors of the virus and human intervention factors may have contributed to its evolution.

The coronavirus disease 2019 (COVID-19) epidemic has posed a huge challenge for healthcare systems worldwide and has had an enormous negative impact on society and the economy (1–4). As the virus evolves, several variants have emerged. For most of 2020, it evolved relatively slowly, which seemed to indicate that vaccines could effectively control infections. However, the first variant of concern (VOC), Alpha was detected in the United Kingdom by December 2020, which suggested that the evolution of SARS-CoV-2 had changed unpredictably. The main driving factors of virus evolution include accidental epidemiological effects, mutations conducive to immune escape and adaptability to new host. In addition, there are occasional low-frequency mutations in the process of virus transmission. A large number of mutations generated in a relatively short time were considered to be triggered by chronic infections of host with lower immune function. Soon after, the second VOC (Beta), the third VOC (Gamma) and the fourth VOC (Delta) were identified in South Africa, Brazil and India successively (Figure 1). VOCs were found in the areas with high infection rate, which is probably related to high mutation frequency promoted by the adaptive evolution of the virus. Human interventions, such as vaccination and prevention and control measures, also have contributed to its evolution. The fifth VOC, Omicron, has the characteristics of both high transmission capacity and antigenic shift, this has driven a new round of global wave. During February to June 2022, the Omicron BA.2 variant was the dominant sublineage (2). This variant has lower virulence, but greater transmissibility, than previous variants. A report from the Jilin province of China suggests that Omicron BA.2 mainly affects the upper respiratory tract, meaning less lung or other organ involvement. Most infected people are asymptomatic or show only mild symptoms; however, a few cases are serious. The propagation rate of BA.2 is high; Dr. Geyu Wang estimates the mean reproductive number (R0) value to be 9.1 (not published). There are few interventions that stop or reduce the spread of disease. The most controversial issue today is whether social isolation or coexistence with the virus is the best way forward.

Here, we present an overview of the evolution of severe acute respiratory syndrome coronavirus 2 (SARS-CoV-2),the pathogen that causes COVID-19, and the characteristics of Omicron and its subvariants BA.1, BA.2, BA.3, BA.4, and BA.5. We also compare Omicron BA.2 with its ancestral SARS-CoV-2 variants, and with influenza viruses, and analyze prevention and control strategies.

## The evolution of SARS-CoV-2

In December 2019, several local health facilities in Wuhan, China, started to report highly contagious pneumonia cases. A new pathogen was isolated from a cluster of these pneumonia patients and named SARS-CoV-2, which shares 89% identical nucleotide sequences with bat SARS-like-CoVZXC21, and 82% with that of human SARS-CoV (3). SARS-CoV-2 spread rapidly throughout the world. The World Health Organization (WHO) declared it a global pandemic on March 11, 2020 (4). To date, the virus has caused epidemics in virtually every country, resulting in more than 490 million confirmed cases and more than 6 million deaths, making it one of the most serious public health disasters in history.

SARS-CoV-2 has undergone a long evolution, and its origin is still unknown. SARS-CoV-2 is a new beta coronavirus (CoV) and belongs to the family *Coronaviridae* and the subfamily *Coronavirinae* (5). Electron micrographs of spike glycoproteins on the virus envelope revealed a crown-like shape; hence it was named coronavirus. Four genera comprise the subfamily: Alpha, Beta, Delta, and Gamma. Genomic characterization suggests that Alpha and beta CoV may originate from rodents and bats, whereas delta and gamma CoV may originate from avian species (4). CoV can cause various diseases in different animals, such as respiratory, intestinal, hepatic and neurological diseases; moreover, it can cross species barriers and cause illness in humans. The manifestations of CoV infection in humans vary from a common cold to severe diseases such as Middle-East Respiratory Syndrome (MERS) and Severe Acute Respiratory Syndrome (SARS). During the course of human history, CoVs have emerged as a major pathogen leading to outbreaks of respiratory disease. MERS-CoV and SARS-CoV are the most virulent and are capable of causing epidemics. The mortality rates of the last SARS-CoV and MERS-CoV epidemics were 10 and 35%, respectively (6). SARS-CoV-2 is a novel beta CoV that contains 4 main structural proteins, 16 nonstructural proteins, and between 5 and 8 accessory proteins. The main structural proteins are the spike protein (S), the nucleocapsid (N), the envelope glycoprotein (E), and the membrane protein (M) (7). Although the origin of SARS-CoV-2 is unknown, genomic comparisons have revealed that SARS-CoV-2 shares high homology (96%) with the beta CoV RaTG13 of bats (8). It is speculated that SARS-CoV-2 transfers from bats to humans via intermediate hosts, such as pangolin (9, 10).

**Figure 2:**
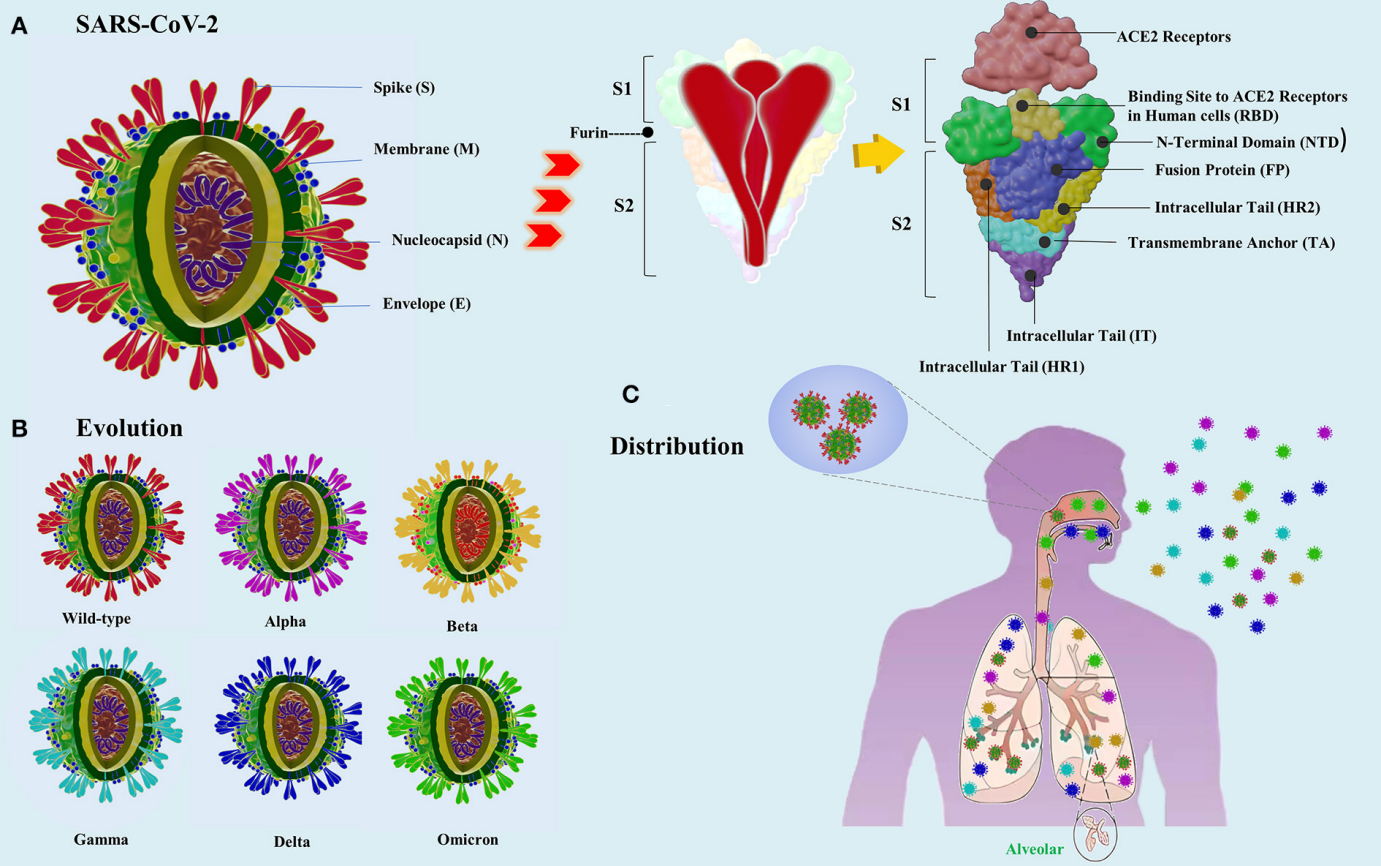
Virus particle structure of SARS-CoV-2 variants. **(A)** Schematic of the original virus [comprising the four main structural proteins spike (S), nucleocapsid (N), envelope (E) glycoprotein, and membrane (M) proteins, which are encoded by a single-stranded RNA genome]. Amplification of the S protein domain is shown, along with its structure. **(B)** Evolution: the main mutations in SARS-CoV-2 variants. The S protein domain of the Alpha, Beta, Gamma, Delta, and Omicron variants harbors 8, 9, 10, 10, and 37 mutations, respectively. There are also mutations in the M, E, and N proteins. **(C)** Distribution of SARS-CoV-2 variants after inhalation into the human respiratory tract. The Omicron variant resides mainly in the upper respiratory tract, whereas the other variants reside mainly in the lower respiratory tract. The Delta variant is found at high levels in the oral cavity.

SARS-CoV-2 has a complex structure. The S glycoprotein, located on the outer surface of the virion, consists of two subunits, S1 and S2 (Figure 2). The primary role of the S1 subunit is to bind to the angiotensin-converting enzyme 2 (ACE2) receptor on the target cell surface and mediate subsequent viral uptake (11). Mutations in the virus receptor-binding domain (RBD) in S glycoprotein of SARS-CoV-2 affect the neutralizing activity of antibodies and vaccine immune sera (12). The S2 subunit is responsible for virus-cell membrane fusion (4, 13). SARS-CoV-2 invades respiratory epithelial cells expressing the ACE2 receptor such as type II alveolar epithelial cells, as well as epithelial cells in other organs (14). Following the viral attachment process, the host transmembrane serine protease 2 (TMPRSS2) generates the S2 subunit, which promotes cell entry by endocytosis, followed by the assembly of the virions (15). The E protein is the most conserved protein and has common characteristics and functions in different coronaviruses (5).

SARS-CoV-2 evolves quickly to adapt to human hosts, and there are multiple variants that may have characteristics different from those of ancestral strains; this is especially true for the S protein and the RBD (16). Initially, the evolution of SARS-CoV-2 was relatively slow until the emergence of the D614G variant, whose increased transmissibility resulted in it becoming the globally dominant variant (17). Since then, the speed at which new variants of SARS-CoV-2 have continued to emerge has steadily increased. Some variants are predicted to cause global pandemics or high mortality and thus have become variants of concern (VOCs). VOCs often have the following characteristics: enhanced transmissibility or virulence, reduced neutralization by natural antibodies or vaccines, enhanced ability to escape detection, and reduced sensitivity to treatments or vaccines. To facilitate in-depth study of these emerging variants as either VOCs or variants of interest, the WHO and CDC have respectively established an independent classification system (4). We are more concerned about VOCs, which have caused new pandemics and thousands of deaths worldwide (18).

## SARS-CoV-2 variants

### Alpha (B.1.1.7)

Thus far, five SARS-CoV-2 VOCs have emerged. The first recognized subtype is Alpha B.1.1.7, previously known as GR/501Y.V1. It was first identified in the United Kingdom at the end of December 2020 (19, 20) and harbors 17 mutations. There are nine mutations (Δ69–70 deletion, Δ144 deletion, N501Y, A570D, P681H, T716I, S982A, D1118H, and D614G) in the S protein. One of them, N501Y, increases the affinity of the S protein for the ACE 2 receptor, thereby enhancing viral adhesion and entry into host cells. B.1.1.7 is 43–82% more transmissible than pre-existing variants and became the dominant variant in the UK at that time (21). A large cohort study of the UK reported that the mortality hazard ratio of B.1.1.7 was 61% (42–82%), which is much higher than that of other variants (22).

### Beta (B.1.351)

The second SARS-CoV-2 VOC is B.1.351, also named the Beta variant or GH501Y.V2, which harbors multiple mutations in the S protein. B.1.351 was first detected in South Africa in October 2020, resulting in the second wave of COVID-19 infections (4). The B.1.351 variant contains nine mutations (L18F, D80A, R246I, K417N, D215G, N501Y, E484K, D614G, and A701V) in the S protein. Three mutations (N501Y, E484K, and K417N) on the RBD of S protein raise its binding affinity for ACE receptors (23–25), which increase the risk of transmission and decrease its susceptibility to neutralization by postvaccination sera, monoclonal antibody therapy, and convalescent sera (4).

### Gamma (P.1)

The Gamma (P.1) variant, also named GR/501Y.V3, was reported in December 2020 in Brazil (26). The B.1.1.28 variant includes ten mutations (L18F, T20N, R190S, H655Y, T1027I V1176, P26S, D138Y, K417T, E484K, and N501Y) in the S protein. Three mutations (N501Y, K417T, and E484K) are located in the RBD, which make this variant less susceptible to neutralization by postvaccination sera, monoclonal antibody therapy, and convalescent sera (27).

### Delta (B.1.617.2)

The fourth VOC was discovered in India in December 2020 and was named Delta (B1.617.2). This variant resulted in this country's most deadly second wave of COVID-19 infection in April 2021, after which it spread rapidly around the world (28). The Delta variant harbors ten mutations (T19R, G142D^*^, 156del, 157del, R158G, L452R, T478K, D614G, P681R, and D950N) in the S protein, although only two are located in the RBD. The Delta variant causes more severe disease than the previous four variants.

### Omicron (B.1.1.529)

On November 24, 2021, the fifth variant was reported in South Africa, and immediately raised global concern because of its high transmissibility (29, 30). The WHO named the variant ‘Omicron' (B.1.1.529). A short time later, the Omicron variant was reported to comprise multiple sublineages: BA.1 (B.1.1.529.1), BA.2 (B.1.1.529.2), BA.3 (B.1.1.529.3), BA.4 (B.1.1.529.4), and BA.5 (B.1.1.529.5). Compared with the Wu-Hanviral genomes of ancestral COVID-19, Omicron BA.1 has up to 60 mutations. Among these, 37 are located in the Protein N-Terminal Domain and RBD regions (31). Compared with BA.1, RBD region of BA.2 is absence of G446S, G496S, and S371L, but has gained D405N, T376A, S371F, and R408S (32). Highly consistent with the RBD of BA.1, the RBD of Omicron BA.3 contains 15 mutation sites, although D405N and S371F in BA.3 replace G496S and S371L in BA.1. Data from Denmark from December 2021 to January 2022 indicated that Omicron BA.2 has increased transmissibility compared to BA.1, and evades vaccine-induced immunity (14, 33). The Omicron BA.2 subvariant quickly replaced Delta and Omicron BA.1, and became a dominant strain worldwide in March 2022 (34, 35). Most Omicron sublineages have an S gene deletion, but BA.2 does not; thus, it cannot be detected by the S-gene target-failure assay and is therefore referred to as the Omicron stealth variant (36). Recently, two new sub-lineages variants BA.4 and BA.5, have been identified. They were discovered in South Africa, and later appeared in Belgium, China, France, Botswana, Germany, Portugal and Australia. The S protein of BA.4 and BA.5 is most closely related to BA.2, except the mutations in BA.2, BA.4, and BA.5 have mutations L452R and 69–70del, wild type amino acid at Q493 and F486V. But the two new variants have been found to be more transmissible and resistant to immunity generated from both infection by previous variants and monoclonal antibodies. D405N and F486V mutations make both BA.2 and BA.4/BA.5 easier to escape from neutralizing antibodies. In South Africa, the BA.4 and BA.5 lineages caused large lower hospital admissions and deaths than previous variants. Cilgavimab and bebtelovimab, which are both therapeutic neutralizing antibodies, can effectively neutralize BA.2.12.1 and BA.4/BA.5, but the S371F, D405N, and R408S mutations undermine most broadly sarbecovirus-neutralizing antibodies. Maybe gene mutation is the result of a survival selection. All the Omicron variants have increased risk of reinfection, and are less susceptible to neutralization by vaccinated and convalescent sera than other SARS-CoV-2 VOCs (16). According to data from the WHO, the symptoms of Omicron infection are not always mild and also may become severe as the disease progresses (Table 1).

**Table 1:** Characteristics and genetic mutations in SARS-CoV-2 variants of concern.

**WHO label**	**Lineage**	**Mutations in RBD of the spike protein**	**Mutations in the rest of the spike protein**	**Impact on transmissibility**	**Impact on vaccine immunogenicity or effectiveness**	**Impact on disease severity**	**References**
Alpha	B.1.1.7	N501Y.	HV69-70Del, Y144Del, A570D, P681H, T716I, S982A, D1118H, D614G.	Yes	No	Yes	(4, 21, 29)
Beta	B.1.351	K417N, E484K, N501Y.	L18F, D80A, D215G, D614G, A701V, R246I.	Yes	Yes	Yes	(4, 29)
Gamma	P.1	K417T, E484K, N501Y.	L18F, T20N, P26S, D138Y, R190S, H655Y, T1027I V1176F.	Yes	Yes	Yes	(4, 27, 29)
Delta	B.1.617.2	L452R, T478K.	T19R, G142D, EF156-157Del, R158G, D614G, P681R, D950N.	Yes	Yes	Yes	(29)
Omicron	BA.1	G339D, S371L, S373P, S375F, K417N, N440K, G446S, S477N, T478K, E484A, Q493R, G496S, Q498R, N501Y, Y505H.	A67V, HV69-70Del, T95I, G142D, VYY143-145Del, N211Del, L212I, Ins214EPE, T547K, D614G, H655Y, N679K, P681H, N764K, D796Y, N856K, Q954H, N969K, L981F.	Yes	Yes	Yes	(4, 29, 37–39)
	BA.2	G339D, S371F, S373P, S375F, T376A, D405N, R408S, K417N, N440K, S477N, T478K, E484A, Q493R, Q498R, N501Y, Y505H.	T19I, LPP24-26Del, A27S, G142D, V213G, D614G, H655Y, N679K, P681H, N764K, D796Y, Q954H, N969K.	Yes	Yes	Yes	(16, 29, 37–39)
	BA.3	G339D, S371F, S373P, S375F, D405N, K417N, N440K, G446S, S477N, T478K, E484A, Q493R, Q498R, N501Y, Y505H.	A67V, HV69-70Del, T95I, G142D, VYY143-145Del, N211Del, L212I, D614G, H655Y, N679K, P681H, N764K, D796Y, Q954H, N969K.	Yes	Yes	Yes	(16, 29, 38)
	BA.4	CG339D, S371F, S373P, S375F, T376A, D405N, R408S, K417N, N440K, S477N, T478K, E484A, Q498R, N501Y, Y505H, L452R, F486V, wild-type amino acid at Q493.	69-70del, T19I, L24S, 25-27del, G142D, V213G, D614G, H655Y, N679K, P681H, N764K, D796Y, Q954H, N969K.	Yes	Yes	Yes	(40)
	BA.5	G339D, S371F, S373P, S375F, T376A, D405N, R408S, K417N, N440K, S477N, T478K, E484A, Q498R, N501Y, Y505H, L452R, F486V, wild-type amino acid at Q493.	69-70del, T19I, L24S, 25-27del, G142D, V213G, D614G, H655Y, N679K, P681H, N764K, D796Y, Q954H, N969K, A1020S.	Yes	Yes	Yes	(40)

## Characteristics of Omicron BA.2

According to clinical data, Omicron BA.2 spreads more quickly than BA.1 but is of similar pathogenicity. The R0 of the Delta variant ranges from 3 to 8 (mean, 5.8) (16); recent studies suggest that the BA.2 strain is about 4·2 and 1·5 times more contagious than Delta and BA.1, respectively (14). Some experts estimate that the R_0_ of Omicron BA.2 is 9·1, suggesting that it is super-infectious and has exponential growth. In addition, Omicron BA.2 is 30% and 17-fold more capable of escaping current vaccines than BA.1 and Delta, respectively (14). Alarmingly, Omicron BA.2 can reinfect individuals who were infected with Omicron BA.1 previously or who have been vaccinated (16). Another study reported positive PCR test results for 86% of nasal swab samples and all saliva swab samples from patients infected with Omicron. Conversely, in patients infected with the Delta variant, only 70% of saliva swabs were positive while all nasal swabs were positive (36). Thus, it is speculated that the high transmission is probably related to the higher levels of Omicron in saliva. Therefore, the variant is more likely to spread when people talk, sing, or cough. Omicron BA. 2 has evolved to several sublineages, BA.2.2, BA.2.2.1, BA.2.12.1, BA.2.13, BA.2.38, BA.2.75, and BA.2.76, and some of them display higher transmissibility than the BA.2. Omicron BA.2 has caused a global pandemic, but at present, there is no published report on geographical distribution difference of Omicron BA.2. To date, we have known that BA.2.12.1 and BA.4/BA.5 exhibit increased evasion of neutralizing antibodies compared with BA.2 in three-dose vaccinated individuals or from individuals who had suffer from a BA.1 infection after vaccination (41). In the future, there may be possible emerging certain recombinants which harbor the BA.2 S gene in a non-BA.2 genomic backbone or other gene mutations, maybe which have different infectivity, pathogenicity, vaccine evasion and the response to treatment.

Additionally, Omicron is often mild, and vaccine tends to be less effective against it. Because Omicron has a reduced ability to induce syncytia in tissue culture, which was investigated as the link with heightened disease severity, the disease of Omicron is less severe, and this has been shown in animal studies. A study based on genome sequence analysis of 4,468 Omicron samples taken from ethnically, geographically, and socioeconomically diverse symptomatic patients indicated that patients infected with Omicron were significantly younger, significantly less likely to be hospitalized, had a significantly shorter median length of hospital stay, and were significantly less likely to need respiratory support than individuals infected with Alpha or Delta (36). Omicron causes significantly more vaccine breakthrough cases than the Delta or Alpha VOCs (55.9, 24.3, and 3.2%, respectively). Another study has revealed that BA.1 is more likely to escape host immune responses than BA.2, so current vaccines, especially the booster vaccine might be more effective against BA.2 (37).

Animal studies demonstrate that the BA. 2 subvariant causes less serious illness than other strains. Kawaoka et al. (32) reported that the pathogenicity and replication of BA.2 are similar to that of BA.1, but are lower than those of ancestral or other variant strains. Both BA.1 and BA.2 infect the alveolar epithelium and bronchioles in the lungs of BALB/c mice; however, lung infections in these mice are much less likely/severe than those caused by the Beta variant. In addition, BA.2 mainly infects the upper respiratory tract, not the lower respiratory tract. The lung proinflammatory cytokine/chemokine response was mild after mice were infected by BA.2. Hence, in animal models, BA.2 variant causes disease of lower severity because of its limited infectivity in the lung. Experiments confirmed that BA.2 replicates to high levels and fuses efficiently in human nasal epithelial cells. But viral replication experiments in hamsters suggest that BA.2 is more pathogenic than BA.1 (42). In addition, some therapeutic monoclonal antibodies have been reported to have lower neutralizing activity against BA.2 than against earlier SARS-CoV-2 variant strains (43, 44).

Many SARS-CoV-2 variants exploit a cell-surface protein, named transmembrane protease serine 2 (TMPRSS2), to infect the cells of lungs and other organs. TMPRSS2 is not expressed on the surface of most cells of the nose and throat. However, Omicron is less likely to wreak havoc in the lungs and does not bind to TMPRSS2 (45). In addition to clinical reports, the WHO has indicated that BA.1 and BA.2 are more likely to infect and reproduce in the upper respiratory tract than Delta, which usually multiplies and infects in the lower respiratory tract. These differences between the Omicron and Delta variants may account for the higher transmissibility of the BA.1 sublineage, as well as lower severity of illness (38, 39). The clinical manifestations of Omicron are also significantly different from those of other strains. A prospective longitudinal observational study analyzed data from the ZOE app (a COVID Symptoms Study App) and compared SARS-CoV-2 infection related symptoms during Delta prevalence and Omicron prevalence. Among 63,002 participants 9,980 patients were enrolled and matched for sex, age and variants prevalent periods. Loss of smell was more common in participants infected during Delta prevalence than during Omicron prevalence (52.7 vs.16.7%, *p* < 0.001). A sore throat was common in both periods, but more common during the period of Omicron prevalence than during the period of Delta prevalence (70.5 vs. 60.8%, *p* < 0.001). The prevalence of symptoms during Omicron infection differed from that during Delta variant infection, apparently with less involvement of the lower respiratory tract and reduced probability of hospital admission in the case of Omicron (1.9 vs. 2.6%, *p* = 0.03) (46).

Almost all positive cases in Jilin Province, China, in March 2022 manifested as upper respiratory tract infection; symptoms included moderate or low fever, dry throat, sore throat, nasal obstruction, runny nose, sneezing, night sweats, fatigue (mild to severe), headache, and drowsiness. In Jilin Province, more than 90% of infected people were asymptomatic or had mild symptoms, but a small number had severe disease. In China (including Hong Kong, Macau, and Taiwan), the mortality rate for subvariant BA2 was 2.393% between February 17, 2022 (the date of the first identification of an Omicron BA.2 case in China) and April 7, 2022, while that on the Chinese mainland was only 0.04% (http://www.nhc.gov.cn/yjb/pqt/new_list_5.shtml). Data from South Africa demonstrate that 78.8% of people infected by Omicron showed mild symptoms or were asymptomatic, while 18.5% needed intensive care, and 2.7% died.

Data from South Africa show that confirmed cases, hospitalizations, and deaths peaked at 117, 63, and 24%, respectively, of the levels reported during the country's Delta wave (29). A French study examined the severity of Omicron BA.2 cases in Marseille between December 27, 2021 and February 14, 2022, and compared it with that reported for 2,793 cases infected by the Omicron BA.1 variant (B.1.1.529.1) (47). Of the BA.2 cases, the median age was 39 years, 54.6% were female, 97.4% had been vaccinated, and 96.9% had been vaccinated with two or three doses. The BA.2 variant of Omicron was significantly more likely to result in hospitalization than the BA.1 variant (6.3 vs. 1.4%, *p* < 0·01). The three patients in the report who died of BA.2 were 80, 97, and 99 years old; two of them had diabetes. The mortality rate was 1.5%. Elderly patients with complications are more likely to succumb to the illness. Other studies also indicate that patients over 60 years old and those with underlying medical conditions (cardiovascular disease, obesity, diabetes, chronic disease) have an increased risk of developing severe COVID-19 infection (4).

## Vaccines effectiveness (VE) against Omicron variants

With the global pandemic of COVID-19, it is hoped to establish herd immunity against SARS-CoV-2 virus through mass vaccination. The vaccine types of COVID-19 include inactivated vaccine, protein subunit vaccine, virus-vectored vaccine, DNA vaccine, mRNA vaccine and so on. Most vaccines target S protein or RBD domain in S protein (48), while the S protein of Omicron variants consists of several mutations, which results in far lower VE against Omicron than other variants. A study reported that mRNA-1273 (Moderna) vaccine and BNT162b2 (Pfizer-BioNTech) vaccine induced neutralizing activities against Omicron were profoundly lower than the neutralization activities against WA1/2020 strain and Beta variant (30-fold reduction of WA1/2020 and 5.7-fold reduction of Beta variant, respectively) (49). About 79% of the subjects were non-responders to Omicron in 2–4 weeks after the second dose vaccine. After 6 months of receiving the second dose, the neutralizing antibodies against Omicron were undetectable in all the subjects who had no prior COVID-19 exposure, which was reversed by third dose vaccine. Another study also proved that the vaccines induced neutralization titers to Omicron declined rapidly within 2 months but restored by boosting vaccine (40). Meta-analysis indicated that the VE against Omicron was 55.9%, which was lowest among the VOCs of COVID-19 (VE against Alpha, Beta, Gamma, Delta is 88.0, 73.0, 63.0, and 77.8%, respectively) (50). Booster vaccination increased VE against Omicron to 80.8%. COVID-19 vaccines are extremely effective in preventing severe disease, hospitalization and death, but they are less effective in preventing infection and mild illness. Clinical data from Southern California showed that COVID-19 vaccination reduced Delta induced cumulative events (per 1,000 cases) of ICU admission, mechanical ventilation and death from 2.0 to 0.3, 1.6 to 0.1, and 1.2 to 0.5, respectively (51). While vaccination mildly reduced the risk of severe outcomes among individuals with Omicron variant infection.

The VE varies among Omicron sublineages. Studies demonstrated that BA.2 resulted in reinfections by escaping from the immunity induced by vaccination and prior infections. The data from Qatar's national COVID-19 databases showed that the vaccine had limited protective effect against BA.1 and BA.2. Qatar has young demographics and high immune basis vaccination rate (exceeding 85%), however, Qatar has been experiencing a large Omicron wave that started on December 19, 2021. Since most of the patients infected with Omicron are asymptomatic or mild, mass scale routine every week RT-qPCR tests in Qatar provides us with more accurate infection data. The data showed that 71.5% of the people infected with Omicron BA.2 subvariant have been vaccinated (52). The main vaccination types in national COVID-19 immunization program in Qatar are BNT162b2 and mRNA-1273 vaccines. The individuals with Omicron BA.2 subvariant infection who were vaccinated had lower RT-qPCR Ct value than those who were unvaccinated, which was similar to the characteristics of BA.1 infected populations. Lower RT-qPCR Ct value represents higher viral load and higher infectivity. This suggests that vaccination does not seem to reduce infectivity. Those who were two dose vaccinated more than 6 months had the lowest RT-qPCR Ct value which suggests that the VE decreased over time. Data from United Kingdom revealed that VE against symptomatic disease with BA.2 was comparable with BA.1 (53). VE against symptomatic disease with BA.1 was 14.8% at 25 weeks or more after two doses either BNT162b2 (Comirnaty, Pfizer–BioNTech), ChAdOx1-S (Vaxzevria, Oxford/AstraZeneca), or mRNA-1273 (Spikevax, Moderna) vaccine, which increased to 70.6% after a week and waned to 37.4% at 15 or more weeks after receiving booster vaccination with either BNT162b2 or mRNA-1273. VE against BA.2 were 27.8, 74.0 and 43.7% in the corresponding period, respectively. A study in the United States found that VE against emergency department/urgent care visits during the BA.2/BA.2.12.2-predominant period was lower than that during the BA.1 period (26 vs. 73%) (54). The same trend was shown for VE against COVID-19–associated hospitalization (61% during BA.1 period vs. 24% during the BA.2/BA.2.12.1 period). The VE against Omicron depends on the sublineages and the types of vaccine. Two dose homologous mRNA vaccines (EUA Moderna mRNA−1273) generated nearly equivalent neutralizing activity against BA.1, BA.2, and BA.3 but modestly reduced neutralizing activity against BA.2.12.1 and BA.4/BA.5 (40).

## Comparison of the characteristics of subvariant BA.2, the ancestral SARS-CoV-2 (2019–2020), and influenza viruses

The outbreak of COVID-19 in the winter of 2019 coincided with the influenza season. During the early stages of the epidemic, SARS-CoV-2 invaded multiple systems and organs, such as spleen, lung, heart, bone marrow, liver, blood vessels, kidneys, gallbladder, and brain tissue. As the virus mutated, the characteristics and virulence changed. The incidence and infection rates are increasing, but the disease is milder, and death rates are falling. Omicron BA.2 infections are mild or asymptomatic, with most symptoms involving upper respiratory discomfort.

**Figure 3:**
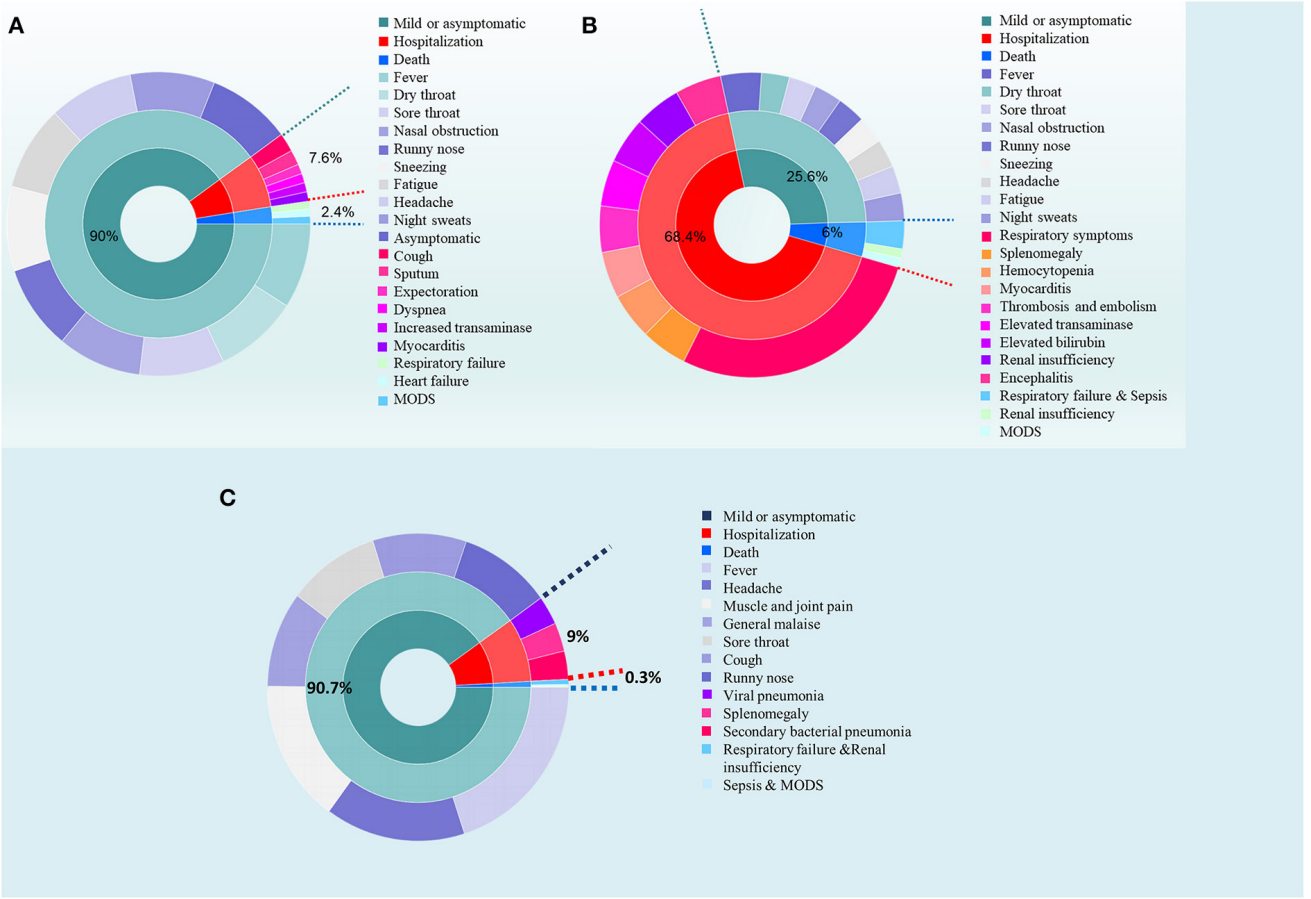
Comparison of clinical symptoms among Omicron BA.2, SARS-CoV-2 and Influenza. **(A)** The harm caused by Omicron BA.2. Most people infected by Omicron BA.2 show symptoms of upper respiratory tract infection; ~90% of cases are asymptomatic and mild, but a small number of cases are severe. In China, the mortality rate for subvariant BA2 is 2.393% (February 17–April 7, 2022). **(B)** The harm caused by SARS-CoV-2 at the early stage of the COVID-19 epidemic. SARS-CoV-2 mainly invades the lungs (i.e., a systemic disease), but can also invade the spleen, bone marrow, heart, blood vessels, liver, gallbladder, kidney, and brain tissue. Almost all patients infected by the original strain and the first four variants required hospitalization. A comprehensive systematic review and meta-analysis of 212 studies comprising 281,461 individuals from 11 countries/regions reported that the rates of severe disease and mortality were about 23 and 6%, respectively. **(C)** The harm caused by influenza. **(A)** Data from Shanghai China show that 96.6% of people infected by influenza **(A)** show mild disease, whereas 2.7% have severe disease and 0.27% die. The rate of hospitalization is 9%. Symptoms include fever, headache, muscle and joint pain, general malaise, sore throat, cough, and runny nose; in the absence of complications, the infection is self-limiting. Complications include pneumonia (viral pneumonia, secondary bacterial pneumonia, and/or secondary fungal pneumonia), nervous system injury, heart damage, myositis, and rhabdomyolysis. MODS, multiple organ dysfunction syndrome.

Milder disease and lower mortality associated with the Omicron subvariants BA.1 and BA.2 have led some experts to consider COVID-19 as a “bad cold” and suggest that society should open up, with no restrictions on social activity; however, this comes at the cost of rapid increases in the number of infections. COVID-19 and influenza differ in many respects. For instance, the diversity and mutation rate of SARS-CoV-2 is half that of the influenza virus (Tables 2, 3) (55, 56). Inequity of vaccine availability and vaccine hesitancy mean that continued epidemics and progression of disease may drive continuous emergence of new variants (Figure 3) (42).

**Table 2:** Differences in the characteristics and epidemiology of the Omicron subvariant BA.2, the ancestral SARS-CoV-2 virus, and influenza viruses.

**Virus**	**Natural hosts**	**Infection mechanism**	**Type**	**R0 (The mean reproductive number)**	**Incubation period (d)**
Omicron BA.2	Bats	S protein interacts with human ACE2** receptors.	RNA	Unclear, some experts estimate 9.1	2–3 d. Infectious during the incubation period; highly contagious within 5 days of onset.
SARS-CoV-2*	Bats	S protein interacts with human ACE2** receptors.	RNA	1.40–6.49 Delta:3–8	Average 4–6 d. Infectious during the incubation period; highly contagious within 5 days of onset.
Influenza viruses	Poultry, wild birds	Respiratory epithelium is the only site for effective cleavage of HA# molecules.	RNA	1.30–1.71	Average 2 d. Contagious from the end of the incubation period until the acute phase.

SARS-CoV-2^*^, severe acute respiratory syndrome coronavirus 2; ACE2^**^, angiotensin converting enzyme 2; HA#, hemagglutinin.

**Table 3:** Differences in the clinical characteristics of Omicron subvariant BA2, COVID-19 (2019–2020), and influenza viruses.

**Disease**	**COVID-19 (Omicron BA.2)**	**COVID-19 (2019–2020)**	**Influenza**
Source of infection	Mainly patients infected with COVID-19.	Patients infected with COVID-19; asymptomatic patients.	Patients and latent infections.
Transmission	Droplets, close contact, contact with infected objects, and airborne (controversial).	Droplets, close contact, contact with infected objects.	Droplets, contact with mucous membranes, contact with infected objects.
Mortality (%)	2.393	6	0.13–1.36
Median age (years)	< 39	44–56	H7N9: 62; H5N1: 26; 2009–H1N1: 25
Sex	Female, 54.6%.	Male, 60%.	Male, 53.8%.
Respiratory symptoms	Dry throat, sore throat, nasal obstruction, runny nose, and drowsiness.	Similar to the common cold in the early stages, nonproductive cough and shortness of breath are relatively common.	Cough.
Other symptoms	Moderate or low fever, neuromuscular issues in old people, a few cases of severe pneumonia.	Fever, chemical sensory disturbance, damage to the reproductive system, constitutional symptoms, and rash.	High fever, conjunctivitis, muscle soreness, a few cases of severe pneumonia.
Hematology	Negative, a few cases of lymphocytopenia.	Lymphocytopenia (47.6%), leukopenia (23.5%), and abnormal liver function tests (14–53%), abnormal renal function (10.9%); thrombocytopenia is uncommon.	White blood cell count is normal or decreased; lymphocytopenia in severe cases Some cases have abnormal liver function tests.
Pulmonary CT	Negative or bronchitis/bronchiolitis; minority have small patches and interstitial changes (more obvious in the periphery); a few serious cases with multiple ground-glass shadows and infiltrates in both lungs.	Multiple small patches and interstitial changes in the early stage (more obvious in the periphery) serious cases have multiple ground-glass shadows and infiltrates in both lungs.	Patchy, ground-glass opacity, and multilobed exudative lesions; cases develop rapidly to diffuse exudation or consolidation in both lungs.
Pathological manifestations	Most show bronchitis/ bronchiolitis; some also have exudation and consolidation in the lungs.	Varying degrees of exudation and consolidation in the lungs and other organ damage; diffuse alveolar damage in 87% of fatal cases.	The ciliated epithelial cells of respiratory tract shed in clusters; the mucosa cells in the lamina propria become hyperemic and edematous, accompanied by mononuclear cell infiltration.
Treatment	Quarantine, general treatment, antiviral therapy, immunotherapy, and traditional Chinese medicine.	Quarantine, general treatment, antiviral therapy, immunotherapy, and traditional Chinese medicine.	Quarantine, general treatment, antiviral therapy, and traditional Chinese medicine.
Prognostic factors of a poor outcome	Age >60; late pregnancy; perinatal period;comorbidities; low immunity; obesity; and heavy smoking.	Age >65; late pregnancy; perinatal period; comorbidities; low immunity; obesity; and heavy smoking.	Age < 5 or ≥65; pregnancy; perinatal period; obesity; comorbidities; and low immunity.

COVID-19, coronavirus disease 2019; CT, computed tomography.

Although Omicron has a lower mortality rate than previous SARS-CoV-2 variants, as well as SARS-CoV in 2002 and the MERS-CoV in 2012, it is much more transmissible. Thus, it can cause large-scale population infections, resulting in a shortage of medical resources, an increase in the number of severe cases and mortality, and negative effects on the economy, politics, and social order. The impact of a virus depends on the proportion of severe cases and the number of infected individuals, which is why influenza kills far more people than Ebola (29). Thus far, prevention of spread has been the strongest and most effective weapon (57).

Current prevention and control measures include vaccination, “one-meter lines,” hand hygiene, wearing masks, extensive diagnostic testing, aggressive contact tracing, and quarantine. Quarantine is one of the most effective tools for controlling communicable disease (57). In the 2 years after onset of the disease, most countries implemented lockdowns, restricting their populations' movements, social gatherings, and general activities to minimize cases. Until now, most of the measures have been consistent, but quarantine is controversial in many countries. China has implemented concurrent prevention strategies to slow transmission of SARS-CoV-2 and minimize cases of severe illness, hospitalization, and death (58). Such strategies include wearing masks; handwashing; avoiding touching the face, mouth, and eyes after interacting with a possibly contaminated environment; improving ventilation; avoiding crowded places and confined spaces; and avoiding close-contact settings. The latter involves closing schools, switching to online education, reducing restaurants' business hours, encouraging working by phone, and curbing the flow of people during vacation (59, 60). Studies indicate that wearing a mask in public effectively reduces the risk of COVID-19 transmission (61, 62).

The Chinese government currently adopts “dynamic zero policy” to control community infection. The process of “dynamic zero policy” is as follows: when positive cases appear, they are isolated, observed, and treated; epidemiological investigation of the case is implemented, and intimate and less-intimate contacts are quarantined in a hotel or a house for several days. Other people in the city stay at home, work at home, and take classes online. Factories, schools, restaurants, and entertainment venues are closed, and buses, taxis, and subways are suspended if necessary. Everyone in the city must undergo nucleic acid testing for 7 consecutive days to identify positive cases as early as possible and curb development of the disease. When the nucleic acid test results are returned, further epidemiological investigations will continue, and intimate and less-intimate contacts will be quarantined. If no new positive cases appear for 7 consecutive days, the government gradually lowers the level of control, and then regions open up gradually. The “dynamic zero policy” reduces the spread of disease significantly, protect elderly patients and children.

In addition, vaccination rates are critical. Universal vaccination programs have been shown to reduce the incidence of severe disease and save lives (63). Yet, vaccines do not confer 100% protection, and some variants (i.e., Omicron and its subvariants) can escape both the vaccine and naturally occurring antibodies; however, vaccination, especially when a booster shot is administered, can protect patients to some extent from becoming critically ill or dying. Vaccine efficacy against symptomatic infection by sublineages BA.1 and BA.2 is 9 and 13%, respectively, for at least 25 weeks following two doses of the vaccine (38, 39); this increases to 63% for BA.1 and 70% for BA.2 at 2 weeks after a third booster dose. In the elderly and those with prior health complications, the mortality rate and disease severity are higher (64). Therefore, all people, including the elderly (≥65 years), are encouraged to get vaccinated.

Previous studies show that early screening, diagnosis and quarantine are effective methods for reducing both the number of people infected and the number of deaths (64, 65). A meta-analysis evaluating 29 studies concluded that quarantine can lower the infection rate from 81 to 44%, and the mortality from 61 to 31% (66). A mathematical model of the spread of COVID-19 in Italy showed that the pandemic could not be controlled and the number of secondary cases would increase in proportion to the size of a household without the strict quarantine rules (57).

Controversially, some retrospective studies report that quarantine has negative psychological effects, including depression, frustration, anxiety, and irritability. Stressors include infection fears, long quarantine duration, inadequate home supplies, boredom, financial loss, inadequate information, and stigma (67). Likewise, quarantine may affect the national economy and quality of life; however, to prevent exponential growth in the number of cases, public health departments must be able to trace contacts of infected cases and reduce their chances of causing further spread. Thus, intrusive action at the early stages of a pandemic might shorten the control time, reduce incidence rates, benefit other fundamental rights, and save both lives and livelihoods (68).

In the beginning, most countries implemented lockdowns to minimize exposure of the general public to potential incoming carriers of COVID-19. However, as the virus mutates and its virulence declines, “opening up” and “coexistence” are considered to be more cost-effective measures. Shockingly, on March 29, 2022, there were 37,085 new cases in the United States, 3,47,374 new cases in South Korea, 2,63,376 new cases in Vietnam, 2,14,392 new cases in the United Kingdom, and 77,797 new cases in Germany. By contrast, 2,333 new cases were reported on the same day in mainland China. So, should infected cases be subjected to quarantine, or should we coexist alongside the virus? There is still a long way to go before we find out which is the better strategy.

## Conclusions

The COVID-19 pandemic has been ongoing for more than 2 years, with multiple variants emerging, the most infectious of which (to date) is Omicron. Although the severe case rate and mortality are low, both are far higher than those associated with influenza. Omicron variants have several mutations in S protein, which result in higher infectivity and lower VE than other variants. The epidemiological and clinical characteristics of BA.2 are worth learning to deal with the new Omicron subvariants. Opening up and coexisting with the virus remains controversial. In the future, more large-scale retrospective studies may provide reliable conclusions.

## Author contributions

RX wrote the manuscript. WW was responsible for data collection and revision of the manuscript. WZ conceived the study and revised the manuscript. All authors contributed to the article and approved the submitted version.

## Funding

This work was supported by grants from the Science and Technology Development Project of Jilin Province (20210401174YY to WZ) and the Natural Science Foundation of Jilin Province (20210101339JC to WW).

## Conflict of interest

The authors declare that the research was conducted in the absence of any commercial or financial relationships that could be construed as a potential conflict of interest.

## Publisher's note

All claims expressed in this article are solely those of the authors and do not necessarily represent those of their affiliated organizations, or those of the publisher, the editors and the reviewers. Any product that may be evaluated in this article, or claim that may be made by its manufacturer, is not guaranteed or endorsed by the publisher.

## References

[1] Sromicki J Schmiady M Maisano F Mestres CA. ECMO therapy in COVID-19: an experience from Zurich. J Card Surg. (2021) 36:1707–12. 10.1111/jocs.15147 33124076

[2] Chen J Wang R Hozumi Y Liu G Qiu Y Wei X Wei GW. Emerging dominant SARS-CoV-2 variants. arXiv [Preprint]. (2022). arXiv: 2210.09485v1. 10.1021/acs.jcim.2c01352 PMC9843632 36577010

[3] Karamitros T Papadopoulou G Bousali M Mexias A Tsiodras S Mendis A. SARS-CoV-2 exhibits intra-host genomic plasticity and low-frequency polymorphic quasispecies. J Clin Virol. (2020) 131:104585. 10.1016/j.jcv.2020.104585 32818852 PMC7418792

[4] Cascella M Rajnik M Aleem A Dulebohn SC Di Napoli R. Features, Evaluation, and Treatment of Coronavirus (COVID-19). StatPearls. Treasure Island, FL: StatPearls Publishing. Copyright © 2022, StatPearls Publishing LLC. (2022). 32150360

[5] Kadam SB Sukhramani GS Bishnoi P Pable AA Barvkar VT. SARS-CoV-2, the pandemic coronavirus: molecular and structural insights. J Basic Microbiol. (2021) 61:180–202. 10.1002/jobm.202000537 33460172 PMC8013332

[6] Wong ACP Lau SKP Woo PCY. Interspecies jumping of bat coronaviruses. Viruses. (2021) 13:11. 10.3390/v13112188 34834994 PMC8620431

[7] Jiang S Hillyer C Du L. Neutralizing antibodies against SARS-CoV-2 and other human coronaviruses. Trends Immunol. (2020) 41:355–9. 10.1016/j.it.2020.03.007 32249063 PMC7129017

[8] Andersen KG Rambaut A Lipkin WI Holmes EC Garry RF. The proximal origin of SARS-CoV-2. Nat Med. (2020) 26:450–2. 10.1038/s41591-020-0820-9 32284615 PMC7095063

[9] Zhang T Wu Q Zhang Z. Probable pangolin origin of SARS-CoV-2 associated with the COVID-19 outbreak. Curr Biol. (2020) 30:1346–51.e2. 10.1016/j.cub.2020.03.022 32197085 PMC7156161

[10] Lam TT Jia N Zhang YW Shum MH Jiang JF. Identifying SARS-CoV-2-related coronaviruses in Malayan pangolins. Nature. (2020) 583:282–5. 10.1038/s41586-020-2169-0 32218527

[11] Song W Gui M Wang X Xiang Y. Cryo-EM structure of the SARS coronavirus spike glycoprotein in complex with its host cell receptor ACE2. PLoS Pathog. (2018) 14:e1007236. 10.1371/journal.ppat.1007236 30102747 PMC6107290

[12] Zhang L Li Q Liang Z Li T Liu S Cui Q. The significant immune escape of pseudotyped SARS-CoV-2 variant Omicron. Emerg Microbes Infect. (2022) 11:1–5. 10.1080/22221751.2021.2017757 34890524 PMC8725892

[13] Aleem A Akbar Samad AB Slenker AK. Emerging Variants of SARS-CoV-2 and Novel Therapeutics Against Coronavirus (COVID-19). StatPearls. Treasure Island, FL: StatPearls Publishing. Copyright © 2022, StatPearls Publishing LLC.; 2022. 34033342

[14] Ziegler CGK Allon SJ Nyquist SK Mbano IM Miao VN Tzouanas CN. SARS-CoV-2 receptor ACE2 Is an interferon-stimulated gene in human airway epithelial cells and is detected in specific cell subsets across tissues. Cell. (2020) 181:1016–35.e19. 10.1016/j.cell.2020.04.035 32413319 PMC7252096

[15] Hoffmann M Kleine-Weber H Schroeder S Krüger N Herrler T Erichsen S. SARS-CoV-2 cell entry depends on ACE2 and TMPRSS2 and is blocked by a clinically proven protease inhibitor. Cell. (2020) 181:271–80.e8. 10.1016/j.cell.2020.02.052 32142651 PMC7102627

[16] Mohapatra RK Kandi V Verma S Dhama K. Challenges of the Omicron (B.1.1.529) variant and its lineages: a global perspective. Chembiochem. (2022) 1:e202200059. 10.1002/cbic.202200059 35322516 PMC9083815

[17] Korber B Fischer WM Gnanakaran S Yoon H Theiler J Abfalterer W. tracking Changes in SARS-CoV-2 spike: evidence that D614G increases infectivity of the COVID-19 Virus. Cell. (2020) 182(4):812-27.e19. 10.1016/j.cell.2020.06.043 32697968 PMC7332439

[18] He X Hong W Pan X Lu G Wei X. SARS-CoV-2 Omicron variant: characteristics and prevention. MedComm. (2020) 2:838–45. 10.1002/mco2.110 34957469 PMC8693031

[19] Galloway SE Paul P MacCannell DR Johansson MA Brooks JT MacNeil A. Emergence of SARS-CoV-2 B.1.1.7 Lineage—United States, December 29, 2020-January 12, 2021. MMWR Morb Mortal Wkly Rep. (2021) 70:95–9. 10.15585/mmwr.mm7003e2 33476315 PMC7821772

[20] Volz E Mishra S Chand M Barrett JC Johnson R Geidelberg L. Assessing transmissibility of SARS-CoV-2 lineage B. 117 in England Nature. (2021) 593:266–9. 10.1038/s41586-021-03470-x 33767447

[21] Davies NG Abbott S Barnard RC Jarvis CI Kucharski AJ Munday JD. Estimated transmissibility and impact of SARS-CoV-2 lineage B.1.1.7 in England. Science. (2021) 372:6538. 10.1126/science.abg3055 33658326 PMC8128288

[22] Davies NG Jarvis CI Edmunds WJ Jewell NP Diaz-Ordaz K Keogh RH. Increased mortality in community-tested cases of SARS-CoV-2 lineage B. 117 Nature. (2021) 593:270–4. 10.1038/s41586-021-03426-1 33723411 PMC9170116

[23] Wibmer CK Ayres F Hermanus T Madzivhandila M Kgagudi P Oosthuysen B. SARS-CoV-2 501Y. V2 escapes neutralization by South African COVID-19 donor plasma. Nat Med. (2021) 27:622–5. 10.1038/s41591-021-01285-x 33654292

[24] Wu K Werner AP Moliva JI Koch M Choi A Stewart-Jones GBE. mRNA-1273 vaccine induces neutralizing antibodies against spike mutants from global SARS-CoV-2 variants. bioRxiv [Preprint]. (2021) 10.1101/2021.01.25.427948 33501442 PMC7836112

[25] Mwenda M Saasa N Sinyange N Busby G Chipimo PJ Hendry J. Detection of B.1.351 SARS-CoV-2 variant strain—zambia, December 2020. MMWR Morb Mortal Wkly Rep. (2021) 70:280–2. 10.15585/mmwr.mm7008e2 33630820 PMC8344984

[26] Faria NR Mellan TA Whittaker C Claro IM Candido DDS Mishra S. Genomics and epidemiology of a novel SARS-CoV-2 lineage in Manaus, Brazil. medRxiv [Preprint]. (2021) 10.1101/2021.02.26.21252554 33853970 PMC8139423

[27] Wang P Casner RG Nair MS Wang M Yu J Cerutti G. Increased resistance of SARS-CoV-2 variant P.1 to antibody neutralization. Cell Host Microbe. (2021) 29:747–51.e4. 10.1016/j.chom.2021.04.007 33887205 PMC8053237

[28] Mlcochova P Kemp SA Dhar MS Papa G Meng B Ferreira I. SARS-CoV-2 B. 16172 Delta variant replication and immune evasion. Nature. (2021) 599:114–9. 10.1038/s41586-021-03944-y 34488225 PMC8566220

[29] Page ML. Understanding omicron. New Sci. (2022) 253:8–9. 10.1016/S0262-4079(22)00030-6 35068646 PMC8759760

[30] Callaway E. Heavily mutated Omicron variant puts scientists on alert. Nature. (2021) 600:21. 10.1038/d41586-021-03552-w 34824381

[31] Kannan S Shaik Syed Ali P Sheeza A. Omicron (B. 11529)—variant of concern - molecular profile and epidemiology: a mini review. Eur Rev Med Pharmacol Sci. (2021) 25:8019–22. 10.26355/eurrev_202112_27653 34982466

[32] Kawaoka Y Uraki R Kiso M Iida S Imai M Takashita E. Characterization and antiviral susceptibility of SARS-CoV-2 Omicron/BA.2. Res Sq. (2022) 1:2 10.21203/rs.3.rs-1375091/v1 35576972 PMC10579982

[33] Fonager J Bennedbæk M Bager P Wohlfahrt J Ellegaard KM Ingham AC. Molecular epidemiology of the SARS-CoV-2 variant Omicron BA.2 sub-lineage in Denmark, 29 November 2021 to 2 January 2022. Euro Surveill. (2022) 27:2200181. 10.2807/1560-7917.ES.2022.27.10.2200181 35272746 PMC8915403

[34] Rahimi F Talebi Bezmin Abadi A. The Omicron subvariant BA. 2: Birth of a new challenge during the COVID-19 pandemic. Int J Surg. (2022) 99:106261. 10.1016/j.ijsu.2022.106261 35167986 PMC8837492

[35] Meo SA Meo AS Al-Jassir FF Klonoff DC. Omicron SARS-CoV-2 new variant: global prevalence and biological and clinical characteristics. Eur Rev Med Pharmacol Sci. (2021) 25:8012–8. 10.26355/eurrev_202112_27652 34982465

[36] Christensen PA Olsen RJ Long SW Snehal R Davis JJ Ojeda Saavedra M. Signals of significantly increased vaccine breakthrough, decreased hospitalization rates, and less severe disease in patients with coronavirus disease 2019 caused by the omicron variant of severe acute respiratory syndrome Coronavirus 2 in Houston, Texas. Am J Pathol. (2022) 192:642–52. 10.1016/j.ajpath.2022.01.007 35123975 PMC8812084

[37] Chen LL Chu AW Zhang RR Hung IF To KK. Serum neutralization of the SARS-CoV-2 omicron sublineage BA.2. Lancet Microbe. (2022) 22:12. 10.1016/s2666-5247(22)00060-x 35373159 PMC8959473

[38] Mahase E. Covid-19: What do we know about omicron sublineages? BMJ. (2022) 376:o358. 10.1136/bmj.o358 35149516

[39] Dhawan M. Priyanka, Choudhary OP. Emergence of Omicron sub-variant BA2: Is it a matter of concern amid the COVID-19 pandemic? Int J Surg. (2022) 99:106581. 10.1016/j.ijsu.2022.106581 35202859 PMC8860472

[40] Lyke KE Atmar RL Islas CD Posavad CM Szydlo D Paul Chourdhury R. Rapid decline in vaccine-boosted neutralizing antibodies against SARS-CoV-2 Omicron variant. Cell Rep Med. (2022) 3:100679. 10.1016/j.xcrm.2022.100679 35798000 PMC9212999

[41] Cao Y Yisimayi A Jian F Song W Xiao T Wang L. BA. 2121, BA4 and BA5 escape antibodies elicited by Omicron infection. Nature. (2022) 608:593–602. 10.1038/s41586-022-04980-y 35714668 PMC9385493

[42] Cheng VC Ip JD Chu AW Tam AR Chan WM Abdullah SMU. Rapid spread of SARS-CoV-2 Omicron subvariant BA.2 in a single-source community outbreak. Clin Infect Dis. (2022). e44–9. 10.1093/cid/ciac203 35271728 PMC8992238

[43] Takashita E Kinoshita N Yamayoshi S Sakai-Tagawa Y Fujisaki S Ito M. Efficacy of antiviral agents against the SARS-CoV-2 Omicron subvariant BA. 2 N Engl J Med. (2022) 386:1475–7. 10.1056/NEJMc2201933 35263535 PMC8929374

[44] Iketani S Liu L Guo Y Liu L Chan JF Huang Y. Antibody evasion properties of SARS-CoV-2 Omicron sublineages. Nature. (2022) 604:553–6. 10.1038/s41586-022-04594-4 35240676 PMC9021018

[45] Meng B Abdullahi A Ferreira IATM Goonawardane N Saito A Kimura I. Altered TMPRSS2 usage by SARS-CoV-2 Omicron impacts infectivity and fusogenicity. Nature. (2022) 603:706–14. 10.1038/s41586-022-04474-x 35104837 PMC8942856

[46] Menni C Valdes AM Polidori L Antonelli M Penamakuri S Nogal A. Symptom prevalence, duration, and risk of hospital admission in individuals infected with SARS-CoV-2 during periods of omicron and delta variant dominance: a prospective observational study from the ZOE COVID Study. Lancet. (2022) 399:1618–24. 10.1016/s0140-6736(22)00327-0 35397851 PMC8989396

[47] Gautret P Hoang VT Jimeno MT Lagier JC Rossi P Fournier PE. The severity of the first 207 infections with the SARS-CoV-2 Omicron BA.2 variant, in Marseille, France, December 2021-February 2022. J Med Virol. (2022) 24:2. 10.1002/jmv.27760 35365865 PMC9088598

[48] Dai L Gao GF. Viral targets for vaccines against COVID-19. Nat Rev Immunol. (2021) 21:73–82. 10.1038/s41577-020-00480-0 33340022 PMC7747004

[49] Edara VV Manning KE Ellis M Lai L Moore KM Foster SL. mRNA-1273 and BNT162b2 mRNA vaccines have reduced neutralizing activity against the SARS-CoV-2 omicron variant. Cell Rep Med. (2022) 3:100529. 10.1016/j.xcrm.2022.100529 35233550 PMC8784612

[50] Zeng B Gao L Zhou Q Yu K Sun F. Effectiveness of COVID-19 vaccines against SARS-CoV-2 variants of concern: a systematic review and meta-analysis. BMC Med. (2022) 20:200. 10.1186/s12916-022-02397-y 35606843 PMC9126103

[51] Lewnard JA Hong VX Patel MM Kahn R Lipsitch M Tartof SY. Clinical outcomes associated with SARS-CoV-2 Omicron (B. 11529) variant and BA1/BA11 or BA2 subvariant infection in Southern California. Nat Med. (2022) 28:1933–43. 10.1038/s41591-022-01887-z 35675841 PMC10208005

[52] Qassim SH Chemaitelly H Ayoub HH AlMukdad S Tang P Hasan MR. Effects of BA.1/BA.2 subvariant, vaccination and prior infection on infectiousness of SARS-CoV-2 omicron infections. J Travel Med. (2022) 29:6. 10.1093/jtm/taac068 35639932 PMC9213851

[53] Kirsebom FCM Andrews N Stowe J Toffa S Sachdeva R Gallagher E. COVID-19 vaccine effectiveness against the omicron (BA. 2) variant in England. Lancet Infect Dis. (2022) 22:931–3. 10.1016/S1473-3099(22)00309-7 35623379 PMC9129256

[54] Link-Gelles R Levy ME Gaglani M Irving SA Stockwell M Dascomb K. Effectiveness of 2, 3, and 4 COVID-19 mRNA vaccine doses among immunocompetent adults during periods when SARS-CoV-2 Omicron BA.1 and BA.2/BA.2.12.1 Sublineages Predominated—VISION Network, 10 States, December 2021–June 2022. MMWR Morb Mortal Wkly Rep. (2022) 71:931–9. 10.15585/mmwr.mm7129e1 35862287 PMC9310634

[55] Piroth L Cottenet J Mariet AS Bonniaud P Blot M Tubert-Bitter P. Comparison of the characteristics, morbidity, and mortality of COVID-19 and seasonal influenza: a nationwide, population-based retrospective cohort study. Lancet Respir Med. (2021) 9:251–9. 10.1016/s2213-2600(20)30527-0 33341155 PMC7832247

[56] Bai Y Tao X. Comparison of COVID-19 and influenza characteristics. J Zhejiang Univ Sci B. (2021) 22:87–98. 10.1631/jzus.B2000479 33615750 PMC7885750

[57] Güner R Hasanoglu I Aktaş F. COVID-19: Prevention and control measures in community. Turk J Med Sci. (2020) 50:571–7. 10.3906/sag-2004-146 32293835 PMC7195988

[58] Rajpal VR Sharma S Kumar A Chand S Joshi L Chandra A. “Is Omicron mild”? Testing this narrative with the mutational landscape of its three lineages and response to existing vaccines and therapeutic antibodies. J Med Virol. (2022) 94:3521–39. 10.1002/jmv.27749 35355267 PMC9088584

[59] Sawakami T Karako K Song P Sugiura W Kokudo N. Infectious disease activity during the COVID-19 epidemic in Japan: Lessons learned from prevention and control measures. Biosci Trends. (2021) 15:257–61. 10.5582/bst.2021.01269 34261848

[60] Sawakami T Karako K Song P. Behavioral changes adopted to constrain COVID-19 in Japan: What are the implications for seasonal influenza prevention and control? Glob Health Med. (2021) 3:125–8. 10.35772/ghm.2021.01066 34250286 PMC8239374

[61] Tabatabaeizadeh SA. Airborne transmission of COVID-19 and the role of face mask to prevent it: a systematic review and meta-analysis. Eur J Med Res. (2021) 26:1. 10.1186/s40001-020-00475-6 33388089 PMC7776300

[62] Darby S Chulliyallipalil K Przyjalgowski M McGowan P Jeffers S Giltinan A. COVID-19: mask efficacy is dependent on both fabric and fit. Future Microbiol. (2021) 16:5–11. 10.2217/fmb-2020-0292 33350330 PMC7784787

[63] Chodick G Tene L Patalon T Gazit S Ben Tov A Cohen D. Assessment of effectiveness of 1 dose of BNT162b2 vaccine for SARS-CoV-2 infection 13 to 24 days after immunization. JAMA Netw Open. (2021) 4:e2115985. 10.1001/jamanetworkopen.2021.15985 34097044 PMC8185600

[64] Sharma A Ahmad Farouk I Lal SK. COVID-19: a review on the novel coronavirus disease evolution, transmission, detection, control and prevention. Viruses. (2021) 13:2. 10.3390/v13020202 33572857 PMC7911532

[65] Iwasaki A Grubaugh ND. Why does Japan have so few cases of COVID-19? EMBO Mol Med. (2020) 12:e12481. 10.15252/emmm.202012481 32275804 PMC7207161

[66] Nussbaumer-Streit B Mayr V Dobrescu AI Chapman A Persad E Klerings I. Quarantine alone or in combination with other public health measures to control COVID-19: a rapid review. Cochrane Database Syst Rev. (2020) 9:Cd013574. 10.1002/14651858.CD013574.pub2 33959956 PMC8133397

[67] Brooks SK Webster RK Smith LE Woodland L Wessely S Greenberg N. The psychological impact of quarantine and how to reduce it: rapid review of the evidence. Lancet. (2020) 395:912–20. 10.1016/s0140-6736(20)30460-8 32112714 PMC7158942

[68] Bauch CT Anand M. COVID-19: when should quarantine be enforced? Lancet Infect Dis. (2020) 20:994–5. 10.1016/s1473-3099(20)30428-x 32445711 PMC7239632

